# Foliar herbivory by caterpillars and aphids differentially affects phytohormonal signalling in roots and plant defence to a root herbivore

**DOI:** 10.1111/pce.13707

**Published:** 2020-01-10

**Authors:** Peter N. Karssemeijer, Michael Reichelt, Jonathan Gershenzon, Joop van Loon, Marcel Dicke

**Affiliations:** ^1^ Laboratory of Entomology Wageningen University and Research Wageningen The Netherlands; ^2^ Department of Biochemistry Max Planck Institute for Chemical Ecology Jena Germany

**Keywords:** above–below‐ground interactions, *Brassica oleracea*, *Delia radicum*, jasmonic acid signalling, plant‐mediated interactions

## Abstract

Plant‐mediated interactions are an important force in insect ecology. Through such interactions, herbivores feeding on leaves can affect root feeders. However, the mechanisms regulating the effects of above‐ground herbivory on below‐ground herbivores are poorly understood. Here, we investigated the performance of cabbage root fly larvae (*Delia radicum*) on cabbage plants (*Brassica oleracea*) previously exposed to above ground herbivores belonging to two feeding guilds: leaf chewing diamondback moth caterpillars (*Plutella xylostella*) or phloem‐feeding cabbage aphids (*Brevicoryne brassicae*). Our study focusses on root‐herbivore performance and defence signalling in primary roots by quantifying phytohormones and gene expression. We show that leaf herbivory by caterpillars, but not by aphids, strongly attenuates root herbivore performance. Above‐ground herbivory causes changes in primary roots in terms of gene transcripts and metabolites involved in plant defence. Feeding by below‐ground herbivores strongly induces the jasmonate pathway in primary roots. Caterpillars feeding on leaves cause a slight induction of the primary root jasmonate pathway and interact with plant defence signalling in response to root herbivores. In conclusion, feeding by a leaf chewer and a phloem feeder differentially affects root‐herbivore performance, root‐herbivore‐induced phytohormonal signalling, and secondary metabolites.

## Abstract 2

Caterpillars (Plutella xylostella) and aphids (Brevicoryne brassicae) feeding on leaves of Brassica oleracea differentially affect gene expression, phytohormone concentrations and defence against cabbage root fly larvae (Delia radicum) in the primary root.

## 1 INTRODUCTION

Most research on insect–plant interactions focusses on what is visible above ground (Kaplan & Denno, 2007, Papadopoulou & van Dam, 2017, Poelman, Broekgaarden, van Loon, & Dicke, 2008, Poelman, Van Loon, Van Dam, Vet, & Dicke, 2010, Stam et al., 2014), yet there is a hidden world beneath our feet, with its own organisms, ecological interactions, food webs, and abiotic environment (Erb, Robert, Hibbard & Turlings, 2011; Johnson et al., 2012; Johnson & Rasmann, 2015; Rasmann et al., 2005). What happens below ground often has major impacts on what we see above ground. For instance, some of the worst agricultural pests are soil dwelling, and they drastically affect plant health (Brown & Gange, 1990; Johnson, Erb, & Hartley, 2016).

However, plants are by no means defenceless. When attacked by insects, plants respond in terms of gene expression, signal transduction via phytohormonal pathways, and eventually responses such as the biosynthesis of secondary metabolites (Erb & Reymond, 2019; Pieterse, Leon‐Reyes, Van der Ent, & Van Wees, 2009). In leaves, chewing herbivores commonly induce a defence response mediated by the jasmonic acid (JA) pathway, whereas phloem feeders usually induce the salicylic acid (SA) pathway (Pieterse, & Does D.V.d., Zamioudis C., Leon‐Reyes A., & Wees S.C.M.V., 2012). Root herbivores induce the JA pathway, although the regulation is different from the above‐ground‐induced JA pathway; however, they seem not to induce the SA pathway (Acosta et al., 2013; Erb, Glauser, & Robert, 2012; Johnson et al., 2016). Defence responses occur not only locally, but throughout the plant. Although most studies on systemic responses focus on above ground tissues, in response to induction in either another leaf or in the roots (Papadopoulou & van Dam, 2017; Soler et al., 2012), there is an increasing body of literature showing that roots respond to leaf herbivory as well (Gulati, Baldwin, & Gaquerel, 2014; Huang, Siemann, Xiao, Yang, & Ding, 2014; Kim, Song, & Ryu, 2016; Kong, Kim, Song, Lee, & Ryu, 2016; Machado et al., 2013; Machado, Arce, McClure, Baldwin, & Erb, 2018; Soler, Erb, & Kaplan, 2013).

Organisms that are spatially separated can interact via such systemic responses, and in this way, the above‐ground and below‐ground communities are linked (Stam et al., 2014). An example of this is induced systemic resistance, in which nonpathogenic rhizosphere microbes enhance defence against above‐ground attackers (Berendsen, Pieterse, & Bakker, 2012; Pieterse et al., 2014; Pineda, Kaplan, & Bezemer, 2017). Insect herbivores also affect each other through such plant‐mediated interactions (Stam et al., 2014). Herbivores feeding on above‐ground plant parts can have a strong impact on root herbivores (Johnson et al., 2012; Soler et al., 2013), but there are large gaps in our understanding of the underlying mechanisms.

The type of defence response, and thus the plant‐mediated effect on subsequent herbivores, that is initiated by a feeding herbivore depends largely on the feeding guild (e.g., chewing or phloem feeding) of the inducing insect (Stam et al., 2014). Chewing herbivores on leaves generally negatively impact root‐feeding insects (Erb, Robert, & Turlings, 2011; Hunt‐Joshi & Blossey, 2005; Johnson et al., 2012), and this has been correlated to changes in secondary metabolites such as tannins or glucosinolates (Huang et al., 2014; Soler et al., 2013). A recent study showed that simulated leaf chewing facilitates the performance of plant parasitic nematodes on roots and that a functional JA pathway is required for this plant‐mediated interaction (Machado et al., 2018). Furthermore, not only direct defence but also the feeding preference of root herbivores (Erb et al., 2015), and attraction of their natural enemies (Rasmann & Turlings, 2007; Soler et al., 2007), can be affected by above‐ground induction. Sap‐feeding herbivores have been shown to induce changes in primary metabolites (Johnson, Hawes, & Karley, 2009), secondary metabolites (Kutyniok & Müller, 2012), root exudation, and recruitment of rhizosphere microbes (Kim et al., 2016). However, the effect of these changes on root herbivores are not consistent; root chewing beetle larvae grew larger on barley plants induced by aphids on leaves (Johnson et al., 2009), but not on Chinese tallow trees (Huang et al., 2014). Conversely, aphids induce resistance against root‐feeding aphids on *Cardamine pratensis* and against root‐feeding nematodes on *Arabidopsis* (Kutyniok & Müller, 2012; Salt, Fenwick, & Whittaker, 1996). The latter was correlated with slight differences in root glucosinolates (Kutyniok & Müller, 2012). Furthermore, above‐ground feeding by whiteflies induced resistance against *Agrobacterium* in roots in an SA‐dependent manner (Song et al., 2015). Thus, the feeding guild of the above‐ground inducer appears to matter for the plant‐mediated effects on root herbivores.

Here, we study how above‐ground insect herbivores with different feeding modes affect the performance of root herbivores and the potential underlying mechanisms. As a study system, we used *Brassica oleracea* plants and their interaction with several specialist insect herbivores. This system has been previously used to study interactions between folivorous insects (Kroes, van Loon, & Dicke, 2015) and transcriptomic responses to various insects on leaves (Kroes et al., 2017, Sarde et al., 2010 in prep). Furthermore, in a closely related plant species, *Brassica nigra*, *Pieris brassicae* caterpillars were found to negatively affect the root‐chewing herbivore *Delia radicum*, the cabbage root fly (Soler et al., 2007). Here, we studied how the chewing herbivore *Plutella xylostella*, the diamondback moth, and the phloem feeder *Brevicoryne brassicae*, the cabbage aphid, affect *D. radicum* in roots. All three species are specialist herbivores of the Brassicaceae family. To shed light on the underlying mechanisms, we examined defence signalling in *B. oleracea* roots. We studied how plants respond to *D. radicum* feeding on the roots, as well as to *P. xylostella* or *B. brassicae* on the leaves. Furthermore, we investigated whether above‐ground herbivory modulates the plant response to root herbivory.

## 2 MATERIALS AND METHODS

### 2.1 Study system

Brussels sprouts plants (*B. oleracea var. gemmifera* cv “Cyrus”) were used for all experiments. Plants were grown in a glasshouse compartment in potting soil (Lentse potgrond, Lent, The Netherlands) at 22 ± 2°C, 50–70% RH, with a 16:8 L:D cycle.

*B. brassicae* L. (Hemiptera: Aphididae) aphids and *P. xylostella* L. (Lepidoptera: Plutellidae) caterpillars were reared on Brussels sprouts plants at 22 ± 2°C, 50–70% RH, with a 16:8 L:D cycle. *D. radicum* L. (Diptera: Anthomyiidae) was collected near Zeewolde, the Netherlands, in 2013 and was reared on swede (*Brassica napobrassica*) at 20 ± 1°C, 50–70% RH, 16:8 L:D cycle.

### 2.2 Root herbivore performance

Three‐week‐old Brussels sprouts plants were infested with 10 *P. xylostella* L1 caterpillars or 10 *B. brassicae* apterous adults. Insects were constrained to the youngest fully expanded leaf (“induced leaf” hereafter) by placing cotton wool around the petiole; this was also done for control plants. In this way, inducing herbivores always started feeding on the same leaf, and most remained on that leaf for the duration of the experiment. Above‐ground inducers were allowed to feed on the leaf for a total of 6 days, after which they were carefully removed with a fine brush. Plants that were cross‐infested or on which removal of above‐ground insects was unsuccessful were removed from the analysis. After 2 days of above‐ground herbivory, 10 *D. radicum* neonate larvae were placed directly on the main root of all plants, just below the soil surface. Plants were distributed over a single greenhouse compartment in blocks to be able to test and correct for spatial differences. All plants received 50 ml of Hyponex (Unifarm, Wageningen, The Netherlands) twice weekly. Plants were watered three times each week. The amount of water given was varied depending on the estimated weight of the pots, as water uptake differs largely depending on the severity of root‐herbivore damage. Twenty days after *D. radicum* induction, plants were individually bagged with mesh nets. From this moment on, plants were checked daily for emerged adults, which were collected and immediately frozen at −18°C. Root fly survival to adulthood was scored, as well as their body weight (Sartorius CP2P micro balance, Germany) and hind tibia length (Dino‐Lite Edge digital microscope, Taiwan).

### 2.3 Gene expression analysis

Induction of plants was carried out as above. Plants were harvested 6 and 24 hr after the start of infestation (hpi) with *D. radicum*. Main roots were cut off using scissors and discs of the induced leaf were collected using a 1‐cm‐diameter cork borer; these tissues were immediately frozen in liquid nitrogen and stored at −80°C. Each sample consisted of three pooled plants.

RNA was extracted using the Bioline Isolate II plant RNA kit (GCbiotech, The Netherlands) according to the manufacturer's instructions. After RNA extraction, cDNA libraries were prepared (SensiFAST™, Bioline). To quantify gene expression, quantitative polymerase chain reaction was performed using SYBR Green (SensiFAST™, Bioline) and primers designed specifically for *B. oleracea* (Table S1). For each tissue type, 10 random samples were analysed for six reference genes (*Act‐2*, *Btub*, *EF1a*, *GAPDH*, *PER4*, and *SAR1a*) to calculate the best combination of reference genes using GeNorm: these were *Btub* and *GAPDH* for leaves, and *Act‐2* and *Btub* for roots (Vandesompele et al., 2002). In leaves, the expression of *LOX2* and *PR1* was assessed. In roots, the transcript levels of *LOX6*, *AOS*, *VSP2*, *MYC2*, *PAL*, *ACS*, *ABA2*, *ORA59*, *PDF1*.*2*, and *PR1* were quantified. Relative expression, normalized to the selected reference genes and the 6‐hr control sample and taking into account primer efficiency, was calculated using the Calibrated Normalized Relative Quantity method in qBase+ version 3.1 (Biogazelle, Zwijnaarde, Belgium).

### 2.4 Phytohormone analysis

From the same samples that were used for gene expression, a portion was lyophilized (Snijders type 2040 lyophylizer, Tilburg, The Netherlands). Phytohormone analysis was performed as in Vadassery et al. (2012) on an Agilent 1200 series HPLC system (Agilent Technologies) with the modification that a tandem mass spectrometer QTRAP 6500 (SCIEX, Darmstadt, Germany) was used. Because it was observed that both the D6‐labelled JA and D6‐labelled JA‐Ile standards (HPC Standards GmbH, Cunnersdorf, Germany) contained 40% of the corresponding D5‐labelled compounds, the sum of the peak areas of D5‐ and D6‐compounds was used for quantification. Concentration of cis‐OPDA and OH‐JA were determined relative to the quantity of the internal standard D6‐JA applying a response factor (RF) of 1.0. OH‐JA‐Ile and COOH‐JA‐Ile were quantified relative to D6‐JA‐Ile: RF 1.0. Sulfo‐JA was determined relative to the quantity of the internal standard D6‐JA: RF 6.0.

### 2.5 Statistics

Differences in gene expression levels and metabolite concentrations between the samples were explored through a multivariate approach, using Partial Least Squares Discriminant Analysis in SIMCA‐P version 15 (Umetrics, Umeå, Sweden). Initial models with all measured variables were used to assess variable importance in projection values. Final models were generated by removing the least important variables (variable importance in projection < 0.75).

All other statistical analyses were carried out in R (R Development Core Team, 2017) using the packages lme4, fitdistrplus, lmtest, and lsmeans (Bates, Mächler, Bolker, & Walker, 2015; Delignette‐Muller & Dutang, 2015; Lenth, 2016; Zeileis & Hothorn, 2002). Distributions were assessed by checking QQ‐plots, histograms, and using the functions shapiro. test, and descdist. Survival of *D. radicum* was analysed using a generalized linear model (GLM) with a Poisson distribution. *D. radicum* development time, weight, and hind tibia length, gene expression levels, and metabolite concentrations were analysed by Generalized Linear Mixed Model using either Gaussian or gamma distributions, with block (position in the greenhouse) as a random factor where relevant. As multiple flies emerged from most plants, for *D. radicum* development time, weight, and hind tibia length, plant was included as a random factor to avoid pseudoreplication. Model selection was done by comparing Akaike Information Criterion values.

## 3 RESULTS

### 3.1 Plant‐mediated effects of above‐ground herbivores on *D. radicum*

**Figure 1:**
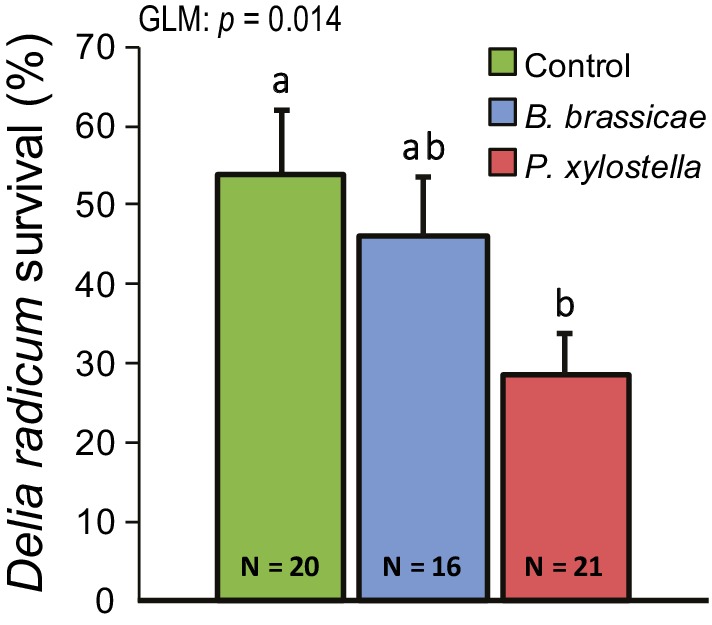
Survival of *Delia radicum* to adulthood on *Brassica oleracea var. gemmifera* plants. Two days prior to *D. radicum* infestation, plants were induced by either *Plutella xylostella* or *Brevicoryne brassicae* on the leaves. Error bars indicate standard errors of the mean. Means having no letters in common differ significantly (Tukey's least significance difference, *p* < .05) [Colour figure can be viewed at http://wileyonlinelibrary.com]

To investigate whether above‐ground herbivory affects *D. radicum* performance on *B. oleracea* roots, a no‐choice experiment was performed (Figure 1). Leaf chewing by *P. xylostella* negatively affected survival to adulthood of *D. radicum* (GLM: *χ* ^*2*^ = 8.55, *df* = 2, *p* = .014), causing a reduction of ca. 43% in survival compared with the control. Survival of root flies following phloem feeding by *B. brassicae* infestation on the leaves was intermediate and not different from survival on either control or *P. xylostella*‐treated plants. Other performance parameters of the flies were unaffected (Figure S1 Development time: GLM: *χ* ^*2*^ = 0.18, *df* = 2, *p* = .92, Weight: LMM: *χ* ^*2*^ = 2.16, *df* = 2, *p* = .34, Tibia length: GLMM: *χ* ^*2*^ = .05, *df* = 2, *p* = .98). This experiment was repeated in a slightly different setup with similar results (Figure S2).

### 3.2 Plant responses to above‐ and belowground herbivory

**Figure 2:**
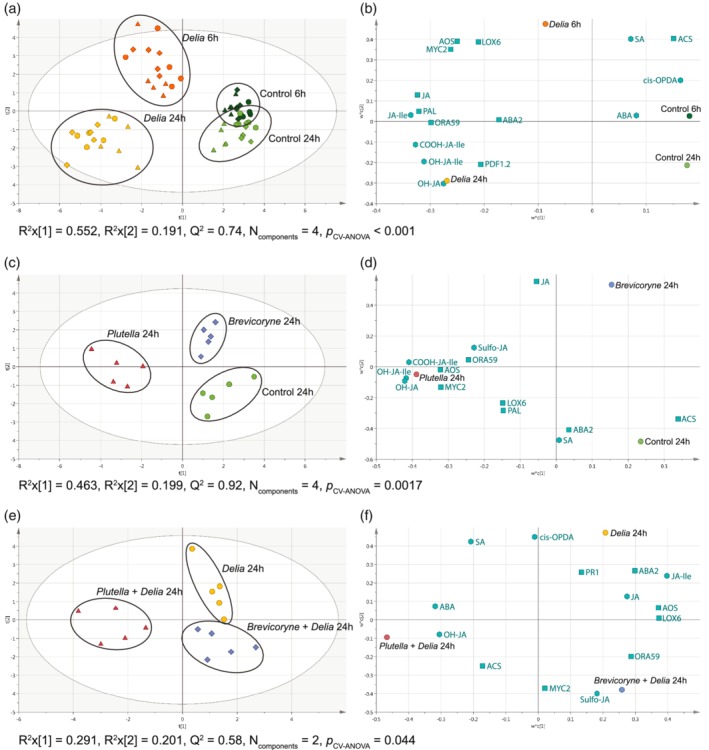
Partial Least Squares Discriminant Analysis illustrating the defence response of *Brassica oleracea* primary roots to *Delia radicum* and two above‐ground herbivores in terms of defence‐related genes and metabolites. Score plots (a, c, e) show separation of samples based on the Partial Least Squares Discriminant Analysis model, loading plots (b, d, f) show the contribution of each gene/metabolite included in the model. The first model (a, b) shows contrasts between plants infested by *D. radicum* for 6 and 24 hr and plants without root herbivory. The second model (c, d) shows differences between the response of primary roots to different above‐ground herbivores in the absence of root herbivory. The third model (e, f) shows how primary roots respond to *D. radicum* in the presence of above‐ground herbivores. Final models were generated by discarding the least important genes/metabolites from full models (VIP < 0.75). Both the second and third models only show the 24‐hr time point. Above‐ground treatments were no above‐ground herbivores, indicated by circles; *Plutella xylostella* larval feeding, indicated by triangles; and *Brevicoryne brassicae* infestation, indicated by diamonds. Grey ellipses in score plots indicate Hotelling's T2 (95%). Black circles delineate treatment groups; they have no statistical value. In loading plots, squares show genes and hexagons show metabolites

Effects of the treatments on gene transcription and metabolite concentrations were assessed through multivariate analyses (Figure 2). In total, transcript levels of 10 genes and concentrations of 9 metabolites involved in plant defence were measured in the primary roots. For all samples together, the first principal component (PC, *R* ^*2*^ = .552), clearly separates samples with and without *D. radicum* (Figure 2a,b; NC = 4, *Q* ^*2*^ = 0.74, *p*_CV‐ANOVA_ < 0.001), indicating that *D. radicum* has a strong effect on the set of genes transcribed and metabolite concentrations. *D. radicum* feeding induced the expression of genes and biosynthesis of metabolites in the jasmonate pathway such as *LOX6*, JA‐Ile, *MYC2* and *ORA59* (Figure 2b). Furthermore, the second PC (*R* ^*2*^ = .191) separates samples taken at six hpi from samples at 24 hpi. To further investigate the effects of the above‐ground treatments, a separate model was built using only samples from the 24‐hre time point without root herbivory (Figure 2c,d; NC = 4, *Q* ^*2*^ = 0.92, *p*_CV‐ANOVA_ = 0.0017). This model shows a separation of the *P. xylostella*‐induced root samples from the other two treatments on the first PC (*R* ^*2*^ = .463). Breakdown products of JA, such as OH‐JA‐Ile, COOH‐JA, and Sulfo‐JA, appear to be important for this separation (Figure 2d). The second PC (*R* ^*2*^ = .199) separates roots of plants induced by *B. brassicae* from control roots. Similar results were obtained when this model was repeated for the 6‐hr time point (Figure S3; NC = 3, *Q* ^*2*^ = 0.8, *p*_CV‐ANOVA_ = 0.0034). Finally, a model was made to explore differences between the *D. radicum*‐induced roots. Here, the first PC separates samples of roots from plants fed upon by *P. xylostella* plus *D. radicum* (*R* ^*2*^ = .291) from the other two treatments (Figure 2e,f); NC = 2, *Q* ^*2*^ = 0.58, *p*_CV‐ANOVA_ = 0.044). JA‐Ile and *ABA2* are associated with roots of plants that were only infested with *D. radicum*, whereas the dual‐infested plants by *P. xylostella* and *D. radicum* are associated with OH‐JA, *ACS*, and ABA. The second PC (*R* ^*2*^ = .201) separates root samples of plants induced by *D. radicum* only from samples induced by both *D. radicum* on roots and *B. brassicae* on leaves. For the 6‐hr samples, no separation was seen between *D. radicum*‐induced roots (Figure S3; NC = 2, Q^2^ = 0.43, *p*_CV‐ANOVA_ = 0.32).

### 3.3 Induction of plant defence by *D. radicum*

**Figure 3:**
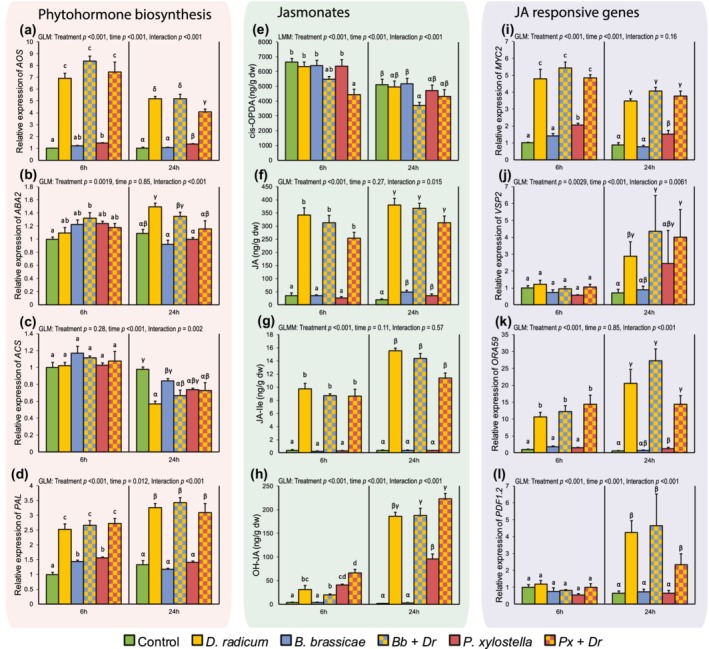
Expression of genes and concentrations of metabolites related to defence signalling in primary roots of *Brassica oleracea var. gemmifera* plants induced by above‐ground (*Brevicoryne brassicae* and *Plutella xylostella*) and below‐ground (*Delia radicum*) insect herbivores. The red panel shows genes related to biosynthesis of defence‐related phytohormones, namely *AOS* (a), *ABA2* (b), *ACS* (c), and *PAL* (d) as markers for biosynthesis of jasmonic acid, abscisic acid, ethylene, and salicylic acid, respectively. The green panel shows concentrations of jasmonate precursor cis‐OPDA (e), bioactive jasmonates JA (f) and JA‐Ile (g), and JA catabolite OH‐JA (h). The blue panel shows genes regulated by JA, transcription fators *MYC2* (i) and *ORA59* (k), and downstream genes *VSP2* (j) and *PDF1.2* (l). Time points indicate time since *D. radicum* induction, plants were infested with above‐ground herbivores 48 hr prior to this. Error bars indicate standard errors of the mean, *N* = 5, each sample represents three pooled plants. Different letters indicate statistically significant differences between treatments within a time point (Tukey's least significance difference, *p* < .05) [Colour figure can be viewed at http://wileyonlinelibrary.com]

Primary roots of plants exhibit a jasmonate response when damaged by *D. radicum* larvae. Jasmonate biosynthesis genes *AOS* and *LOX6* were upregulated by *D. radicum* feeding (Figure 3a, Figure S4). The bioactive jasmonates JA and JA‐Ile were strongly induced following *D. radicum* herbivory (Figure 3f). Compared with control, JA increased 10‐fold and 20‐fold, whereas JA‐Ile increased 25‐fold and 42‐fold, after 6 and 24 hr, respectively. Further downstream, the JA‐related transcription factors *MYC2* and *ORA59* were induced by *Delia* herbivory (Figure 3i,k), and after 24 hr of feeding, *VSP2* and *PDF1.2*, two genes encoding defence proteins, were activated (Figure 3j,l).

In addition to the jasmonate pathway, other hormonal pathways also changed in response to *D. radicum* feeding. The phenylpropanoid pathway marker *PAL* was activated in plants infested by *D. radicum* (Figure 3d), but SA levels were unchanged (Figure S3). After 24 hr of *Delia* feeding, primary roots had decreased *ACS* transcription, indicating lower ET biosynthesis (Figure 3c), whereas *ABA2*, an ABA biosynthesis gene, was upregulated (Figure 3b). Conversely, there was a trend for lower ABA hormone levels 24 hr after root herbivore induction compared with control roots (Figure S3; Tukey's least significance difference; *z* = 2.79, *p* = .058).

### 3.4 Effects of *P. xylostella* on primary root defence signalling

Folivory by *P. xylostella* systemically enhanced defence responses in the primary roots. Transcription of *AOS*, involved in biosynthesis of JA, was slightly upregulated relative to control plants in response to caterpillar feeding on leaves (Figure 3a). Indeed, JA levels were slightly increased at the 24‐hr time point (72 hr after *P. xylostella* induction) compared with control samples (Figure 3f). The jasmonate‐regulated transcription factors *MYC2* and *ORA59* were also expressed at higher levels in *Plutella*‐induced roots compared with control (Figure 3i,k). However, compared with control plants, none of the active components of the JA pathway were increased as much by *P. xylostella* as they were by local induction by *D. radicum* (Figure 3). Interestingly, inactive jasmonates (OH‐JA, OH‐JA‐Ile, and COOH‐JA‐Ile) accumulated in the primary roots of *Plutella*‐infested plants (Figure 3h, Figure S4).

### 3.5 Effects of *B. brassicae* on primary root defence signalling

Above‐ground feeding by aphids had little effect on the jasmonate pathway in the primary roots. Aside from a slight increase in JA levels at the 24‐hr time point (72 hr after aphid infestation) relative to control roots, no other markers were changed in response to *B. brassicae* infestation on the leaves (Figure 3). However, at the 6‐hr time point (54 hr after the start of aphid induction), *PAL* expression was upregulated following *B. brassicae* treatment compared with control roots, and a *PR1* response was seen (Figure 3d, Figure S4). Root SA concentrations were not altered by above‐ground *B. brassicae* feeding (Figure S4). Interestingly, the SA pathway marker gene *PR1* was unaffected in local tissues where aphids fed (Figure S5), even though several colonies had formed on each induced leaf by the time of harvest.

### 3.6 Interactive effects between above‐ and below‐ground inducers on root defence signalling

The plant response to *D. radicum* was altered when plants had previously been infested with above‐ground herbivores. When both *D. radicum* and *P. xylostella* were present, *AOS* and *LOX6* were downregulated after 24 hr compared with plants that were only induced by *D. radicum*, implying lower jasmonate biosynthesis rates in these roots (Figure 3a, Figure S4). Levels of cis‐OPDA in the root were lower in plants exposed to dual herbivory, whereas single herbivore treatments did not affect the concentration of this jasmonate precursor (Figure 3e). Expression levels of downstream genes in the JA cascade, *MYC2*, *ORA59*, *PDF1.2*, and *VSP2*, did not differ between plants induced by *D. radicum* only and dual‐infested plants (Figure 3i–l). Interestingly, the upregulation of *ABA2* 24 hr after *Delia* induction was not found when *P. xylostella* was present on the plants (Figure 3b). Finally, although SA hormone concentrations were not affected by aphids alone, the combination of *B. brassicae* and *D. radicum* caused a decrease in this signalling compound relative to control roots (Figure S4).

## 4 DISCUSSION

**Figure 4:**
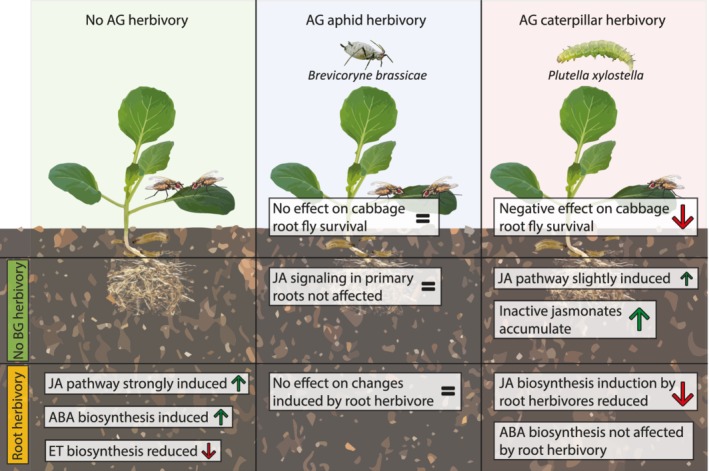
Overview of the effects of above ground herbivory by aphids (*Brevicoryne brassicae*) or caterpillars (*Plutella xylostella*) on root herbivore (*Delia radicum*) survival and primary root defence signalling. A distinction is made between defence signalling induced by above‐ground herbivores alone, and how above‐ground herbivores affect the plant response to root herbivores. AG, above ground; BG, below ground; JA, jasmonic acid; ABA, abscisic acid; ET, ethylene [Colour figure can be viewed at http://wileyonlinelibrary.com]

Our data show that leaf herbivory has a strong effect on root herbivores, and that this effect is dependent on the feeding guild of the above‐ground attacker (Figure 4). We show that leaf herbivory causes changes in transcript levels and signalling compounds in primary roots. In particular, we show that the jasmonate pathway is induced by root herbivores and that above‐ground herbivores induce changes in this pathway in the roots, which may underlie the plant‐mediated interaction described here. Furthermore, above‐ground herbivores interact with defence induction by root herbivores, leading to a different signal signature in the primary root.

Responses of plants to *D. radicum* involve a strong activation of jasmonates. The JA pathway is well‐known for regulating defence against chewing herbivores, both in leaves and roots; in rice, mutants lacking a functional JA response were more susceptible to root herbivores (Erb & Reymond, 2019; Lu et al., 2015). Furthermore, both root herbivory and jasmonate treatment triggers maize roots to produce of a volatile compound that attracts entomopathogenic nematodes (Erb et al., 2011; Rasmann et al., 2005). The magnitude of jasmonate induction by *D. radicum* is quite surprising, as Erb et al. (2012) reported that many plant species lack a strong jasmonate burst in their roots, instead relying on a more subtle increase compared with leaves. In leaves of the same cultivar as we use here, the magnitude of JA induction after 24 hr of feeding by several caterpillar species was shown to lie between 4‐fold and 11‐fold (Bruinsma et al., 2009; Bruinsma et al., 2010), much less compared with the 20‐fold increase we find in primary roots responding to root‐feeding maggots. Possibly, this is due to our focus on primary roots, whereas to the best of our knowledge, previous studies did not distinguish between root tissues in terms of jasmonate concentrations. Root tissues that have a higher value in terms of plant fitness have higher levels of chemical defences in *Brassica* species and maize, in line with the optimal defence theory (Robert et al., 2012; Tsunoda, Krosse, & van Dam, 2017). Possibly this is also true for the high inducibility of jasmonates in primary roots found here.

In addition to the JA pathway, *D. radicum* induced changes in the expression of ABA and ET biosynthesis genes after 24 hr, suggesting that these hormones play a role in later stages of the defence response. Because the symptoms of root herbivory by *Delia* resemble those of drought, involvement of ABA is not surprising, as it is the main regulator of abiotic stress resistance (Finkelstein, Gampala, & Rock, 2002). Furthermore, ABA was shown to play a role in the response of maize to the root herbivore *Diabrotica virgifera virgifera* (Erb et al., 2009). Moreover, from studies on leaf defence signalling, we know that ABA and ET are important in fine‐tuning JA responses (Pieterse et al., 2009). In above‐ground tissues, MeJA induction regulates over 3,500 transcripts in *Arabidopsis thaliana* (Hickman et al., 2017), and onion thrips (*Thrips tabaci*) feeding influences the transcription of about 10% of all *B. oleracea* genes (Sarde et al., 2010 in prep). Not all of these are involved in defence, as JA regulates many other processes, such as the regulation of root growth, formation of root hairs, lateral roots, and adventitious roots (Wasternack & Feussner, 2018). Indeed, activation of the jasmonate cascade does not always lead to enhanced defence. Exogenous jasmonate treatment of the root caused a decline in *Delia* pupation in broccoli plants, *B. oleracea*, but had the opposite effect in turnip, *Brassica rapa* (Pierre et al., 2012). Possibly, ABA and ET in roots fine‐tune the JA response to a specific subset of genes.

There is ample evidence that chewers feeding on leaves negatively affect chewers feeding on roots, which is in line with our findings (Erb et al., 2011; Johnson et al., 2012; Masters & Brown, 1992; Soler et al., 2007). Although survival of root chewers is usually reduced in these interactions, growth is often increased, which may lead to some level of compensation (Johnson et al., 2012). Here, however, other performance parameters of root chewers were unchanged by above‐ground herbivory, so the surviving *D. radicum* individuals did not benefit from reduced competition. Several mechanisms have been proposed to explain these plant‐mediated interactions. Primary metabolism seems a likely candidate for mediating interactions between above‐ and below‐ground herbivores, as tolerance is thought to be achieved by plants that allocate their resources in roots upon leaf attack (Schwachtje & Baldwin, 2008). Indeed, leaf herbivory has been found to increase allocation of resources to roots (Holland, Cheng, & Crossley, 1996; Schwachtje et al., 2006). On the other hand, carbohydrate storage decreases in roots following leaf herbivory (Machado et al., 2013). Others have pointed to increased secondary metabolites as the main mediators of antagonism between above‐ and below‐ground chewers (Soler et al., 2013). A well‐documented example of this is found in Chinese tallow trees (*Triadica sebifera*), on which leaf chewers negatively affected flea beetle larvae in the roots, but conspecific adult beetles feeding on the leaves did not. In this system, root tannin concentrations in the different treatments correlated with the performance of the root herbivores (Huang et al., 2014). In *B. oleracea*, an increase in indole glucosinolates was recorded in roots of plants challenged by *Phyllosticta* *brassicae* caterpillars above ground, which was suggested to play a role in a negative effect on *D. radicum* (Soler et al., 2007). However, whether these toxins provide defence against the specialist *D. radicum* is debatable, because glucosinolates did not correlate with *D. radicum* performance in several studies (Pierre et al., 2012; Van Geem, Harvey, Cortesero, Raaijmakers, & Gols, 2015). Furthermore, *D. radicum* harnesses gut microbes that can disarm toxic isothiocyanates resulting from the breakdown of gluconasturtiin, an aromatic glucosinolate (Welte et al., 2015), and it may well possess methods to detoxify aliphatic and indolic glucosinolates as well. Research on *A. thaliana* has shown that flavonoids rather than glucosinolates are involved in defence against specialist insects (Onkokesung et al., 2014; Onkokesung et al., 2019). To understand the mechanism underlying the interaction between *P. xylostella* and *D. radicum*, more components of root defence (e.g., secondary metabolites and defensive proteins) should be investigated, and manipulative approaches should be used.

Above‐ground infestation by *P. xylostella* causes changes in root defence signalling. In maize, above‐ground caterpillar feeding failed to induce jasmonate levels in roots (Erb et al., 2009), whereas in tobacco, an increase in root jasmonates is recorded 2 hr after the application of leaf damage plus caterpillar oral secretion (Machado et al., 2018). Indeed, a functional jasmonate pathway was needed to allow plant‐mediated facilitation of above‐ground‐simulated herbivory on nematodes in tobacco roots (Machado et al., 2018). Here, we report a slight increase in JA levels in roots following leaf herbivory. A small increase may, however, have a large impact in roots (Erb et al., 2012). Furthermore, we find an increase in genes encoding enzymes catalyzing JA biosynthesis and downstream transcription factors. Among the differences, roots of plants infested with *P. xylostella* on the leaves harboured much higher levels of jasmonate derivatives that are mostly inactive in signal transduction (Wasternack & Hause, 2013). Accumulation of jasmonate derivatives indicates that a jasmonate response occurred before our measurements started. This earlier jasmonate response could have led to more defensive metabolites, or could have primed plant defence in the roots, enabling the plant to respond more rapidly to *D. radicum*. Interestingly, some JA derivatives may retain partial activity; for instance, OH‐JA treatment leads to slight induction of JA‐related marker genes in *Arabidopsis* and a faster induction by JA‐Ile treatment when applied together (Smirnova et al., 2017). Furthermore, recently, the inactive OH‐JA‐Ile was synthetically reactivated by modifications that can theoretically occur in nature, and these reactivated compounds can activate defence against *Manduca sexta* caterpillars (Jimenez‐Aleman, Machado, Baldwin, & Boland, 2017; Jimenez‐Aleman, Machado, Görls, Baldwin, & Boland, 2015).

In addition to altering the basal levels of defence in systemic tissues, plant‐mediated interactions can involve defence priming, in which the induced response is altered because of a previous event (Erb, Ton, Degenhardt, & Turlings, 2008). Indeed, plants previously infested by *P. xylostella* responded differently to *D. radicum*. Transcripts of JA biosynthesis genes were less abundant in coinfested plants compared with plants only infested by *D. radicum*. This reduction could be the result of negative feedback in the jasmonate pathway, suggesting an earlier plant response to *D. radicum* when *P. xylostella* was already present (Chini et al., 2007; Liu et al., 2019). Alternatively, *D. radicum* may have fed less or died early on roots of *P. xylostella* induced plants. In maize plants, root herbivores were shown to avoid plants that were previously induced by caterpillars (Erb et al., 2015). However, because *D. radicum* tunnels through the tap root of cabbage plants, their feeding behaviour is hard to observe, especially in the early stages of infestation. Interestingly, although *D. radicum*‐infested roots contained higher levels of *ABA2* transcripts at 24 hr, this was attenuated when *P. xylostella* was present. It is tempting to suggest that fine‐tuning differences within the JA pathway, or differences in other ABA‐regulated genes, may play a role in the plant‐mediated interaction between *P. xylostella* and *D. radicum*. To investigate this further, a transcriptomic approach with more time points is required.

Plant‐mediated interactions between different feeding guilds are rarely studied, in particular the effects of above‐ground phloem‐feeding insects on below‐ground chewers. In *T. sebifera*, aphids had no effect on root‐feeding flea beetle larvae (Huang et al., 2014). In barley, aphids did not affect survival of root‐feeding wireworm larvae, but positively influenced their growth (Johnson et al., 2009). In line with these two studies, the effect of aphids on *D. radicum* in our study was weak. Interestingly, other below‐ground feeders are more strongly affected by above‐ground induction. For instance, *B. brassicae* negatively affected plant‐parasitic nematode performance in roots of *A. thaliana* (Kutyniok & Müller, 2012), and on *Cardamine pratensis*, leaf feeding aphids negatively affected root‐feeding aphids (Salt et al., 1996). In above‐ground tissues, phloem‐feeding insects and chewers have been shown to facilitate one another (Soler et al., 2012). The finding that this does not occur between foliar aphids and root‐feeding insects may indicate that mechanisms underlying these interactions do not travel into the roots.

Although the plant‐mediated effects of aphids on below‐ground chewers may be weak or absent, this does not necessarily indicate a lack of induction of below‐ground defence. Systemic effects of aphids from leaves to roots have been reported in terms of primary metabolites (Johnson et al., 2009; Masters & Brown, 1992), secondary metabolites (Kutyniok & Müller, 2012), and root exudates (Kim et al., 2016). Another phloem‐feeding hemipteran, *Bemisia tabaci*, induces genes involved in biosynthesis of jasmonates and anthocyanins of maize roots (Park, Bae, & Ryu, 2015). In our study, however, above‐ground infestation of aphids had little effect on the measured root defence markers. It is quite possible that we missed changes induced by the aphids, because we focussed mainly on markers in the jasmonate defence pathway. On the other hand, SA levels and *PR1* transcripts in roots did not exhibit a strong aphid response either. Aphid‐induced effects can be highly density dependent (Kroes et al., 2015), perhaps a higher initial number of aphids would yield a different result. The differences induced by aphids that we observed, such as a slight increase in *PAL* transcripts, as well as changes we may have missed, did not change plant defence against *D. radicum* at the aphid density we studied.

*D. radicum* appears to elicit a suboptimal defence response in their host plants, because induction by *P. xylostella* leads to much more effective defence. Herbivorous insects are known to be able to manipulate their host's immune system by using effectors in their saliva (Acevedo, Rivera‐Vega, Chung, Ray, & Felton, 2015; Consales et al., 2012) or even by symbiosis with microorganisms (Chung et al., 2013; Kazan & Lyons, 2014; Ziebell et al., 2011), leading to induced susceptibility. For instance, Colorado potato beetle (*Leptinotarsa decemlineata*) larvae use bacteria in their saliva to trick their host plant into an SA‐based defence response (Chung et al., 2013). Root herbivores can also cause induced susceptibility, for example, *D. v. virgifera* aggregate on maize roots and facilitate each other in a plant‐mediated manner (Robert et al., 2012). It is unknown whether *D. radicum* possesses a similar mechanism, although it seems feasible, especially because *D. radicum* shows aggregated distributions in cabbage fields (Mukerji & Harcourt, 1970), prefers to oviposit on conspecific‐damaged plants (Robert, Vladimír, Bruno, & Erich, 1996), and also performs better on plants previously damaged by conspecifics (Pierre et al., 2012). A targeted search for host‐manipulation mechanisms by *D. radicum* is likely to provide insights into the evolutionary arms race between brassicaceous plants and these specialist root‐feeding herbivores.

## 5 CONCLUSION

The current study shows that above‐ground herbivores, depending on the species, can influence root herbivores. We show that above‐ground herbivory influences not only the basal defence but also root‐herbivore induced defence in primary roots. Research on interactions between above‐ and below‐ground herbivory improves the understanding of plants as a whole organism. This can help not only in breeding for better crops but also to better understand ecological processes in nature, where plants are always dealing with multiple stressors in multiple organs.

## Supporting information


**Figure S1**. Development time from neonate to adult (a), adult weight (b) and adult hind tibia length (c) of *Delia radicum* on *Brassica oleracea* var. *gemmifera* plants. Two days prior to *D. radicum* infestation, plants were induced by either *Plutella xylostella* or *Brevicoryne brassicae* on the leaves. Error bars indicate standard errors of the mean.
**Figure S2**. Survival of *Delia radicum* flies to adulthood on *Brassica oleracea* var. *gemmifera* plants. Prior to D. radicum infestation, plants were induced by either *Plutella xylostella* or *Brevicoryne brassicae*. Methods similar as described in material and methods, with two exceptions: aboveground inducers were placed on the plant 7 days before *D. radicum* and left for 6 days, and 10 *D. radicum* larvae were used. Error bars indicate standard errors of the mean. Different letters indicate statistically significant differences (Tukey's LSD, p < 0.05).
**Figure S3**. PLS‐DA analyses illustrating the defence response of *Brassica oleracea* primary roots to *Delia radicum* and two aboveground herbivores in terms of defence related genes and metabolites. Score plots (a,c,e) show separation of samples based on the PLS‐DA model, loading plots (b,d,f) show the contribution of each gene/metabolite included in the model. The first model (a,b) shows differences between the response of primary roots to different aboveground herbivores in the absence of root herbivory. The second model (c,d) shows how primary roots respond to *D. radicum* in the presence of aboveground herbivores. Final models were generated by discarding the least important genes/metabolites from full models (VIP < 0.75). Both models were made using only one time point, 6 h after *D. radicum* infestation. Aboveground treatments are indicated by shapes, circles: no aboveground herbivores, triangles: *Plutella xylostella*, diamonds: *Brevicoryne brassicae*. Grey ellipses in score plots indicate Hotelling's T2 (95%). Black circles delineate treatment groups, they have no statistical value. In loading plots, squares show genes and hexagons show metabolites.
**Figure S4**. Expression of genes and concentrations of metabolites related to defence signalling in primary roots of *Brassica oleracea* var. *gemmifera* plants induced by aboveground (*Brevicoryne brassicae* or *Plutella xylostella*) and belowground (*Delia radicum*) insect herbivores. Time points indicate time since *D. radicum* induction, plants were infested with aboveground herbivores 48 h prior to this. Error bars indicate standard errors of the mean, N = 5, each sample represents 3 pooled plants. Different letters indicate statistically significant differences between treatments within a time point (Tukey's LSD, p < 0.05).
**Figure S5**. Expression of *LOX2* (a) and *PR1* (b) in leaves of *Brassica oleracea* var. *gemmifera* plants induced by aboveground (*Brevicoryne brassicae* and *Plutella xylostella*) and belowground (*Delia radicum*) insect herbivores. Samples were taken at the site of leaf damage. Time points indicate time since *D. radicum* induction, plants were infested with aboveground herbivores 48 h prior to this. Error bars indicate standard errors of the mean, N = 5, each sample represents 3 pooled plants. Different letters indicate statistically significant differences between treatments within a time point (Tukey's LSD, p < 0.05).
**Table S1**. Primers used for qPCR analyses of *Brassica oleracea* roots and leaves.
