# Supplementary material for: Foliar herbivory by caterpillars and aphids differentially affects phytohormonal signalling in roots and plant defence to a root herbivore
Source: Plant Cell Environ. 2020 Jan 10;43(3):775–86. doi: 10.1111/pce.13707 (PMC7065167; doi:10.1111/pce.13707)
Supplement: Supplementary file 1 — Figure S1. Development time from neonate to adult (a), adult weight (b) and adult hind tibia length (c) of Delia radicum on Brassica oleracea var. gemmifera plants. Two days prior to D. radicum infestation, plants were induced by either Plutella xylostella or Brevicoryne brassicae on the leaves. Error bars indicate standard errors of the mean. Figure S2. Survival of Delia radicum flies to adulthood on Brassica oleracea var. gemmifera plants. Prior to D. radicum infestation, plants were induced by either Plutella xylostella or Brevicoryne brassicae. Methods similar as described in material and methods, with two exceptions: aboveground inducers were placed on the plant 7 days before D. radicum and left for 6 days, and 10 D. radicum larvae were used. Error bars indicate standard errors of the mean. Different letters indicate statistically significant differences (Tukey's LSD, p < 0.05). Figure S3. PLS‐DA analyses illustrating the defence response of Brassica oleracea primary roots to Delia radicum and two aboveground herbivores in terms of defence related genes and metabolites. Score plots (a,c,e) show separation of samples based on the PLS‐DA model, loading plots (b,d,f) show the contribution of each gene/metabolite included in the model. The first model (a,b) shows differences between the response of primary roots to different aboveground herbivores in the absence of root herbivory. The second model (c,d) shows how primary roots respond to D. radicum in the presence of aboveground herbivores. Final models were generated by discarding the least important genes/metabolites from full models (VIP < 0.75). Both models were made using only one time point, 6 h after D. radicum infestation. Aboveground treatments are indicated by shapes, circles: no aboveground herbivores, triangles: Plutella xylostella, diamonds: Brevicoryne brassicae. Grey ellipses in score plots indicate Hotelling's T2 (95%). Black circles delineate treatment groups, they have no statistical value. In loa [file PCE-43-775-s001.docx]

Supporting Information

# Article title

Foliar herbivory by caterpillars and aphids differentially affects phytohormonal signalling in roots and plant defence to a root herbivore

# Authors

Peter N. Karssemeijer^1^, Michael Reichelt^2^, Jonathan Gershenzon^2^, Joop van Loon^1^, Marcel Dicke^1^

1. Laboratory of Entomology, Wageningen University and Research, Droevendaalsesteeg 1, 6708PB Wageningen, The Netherlands

2. Department of Biochemistry, Max Planck Institute for Chemical Ecology, Hans-Knoell-Str. 8, 07745, Jena, Germany


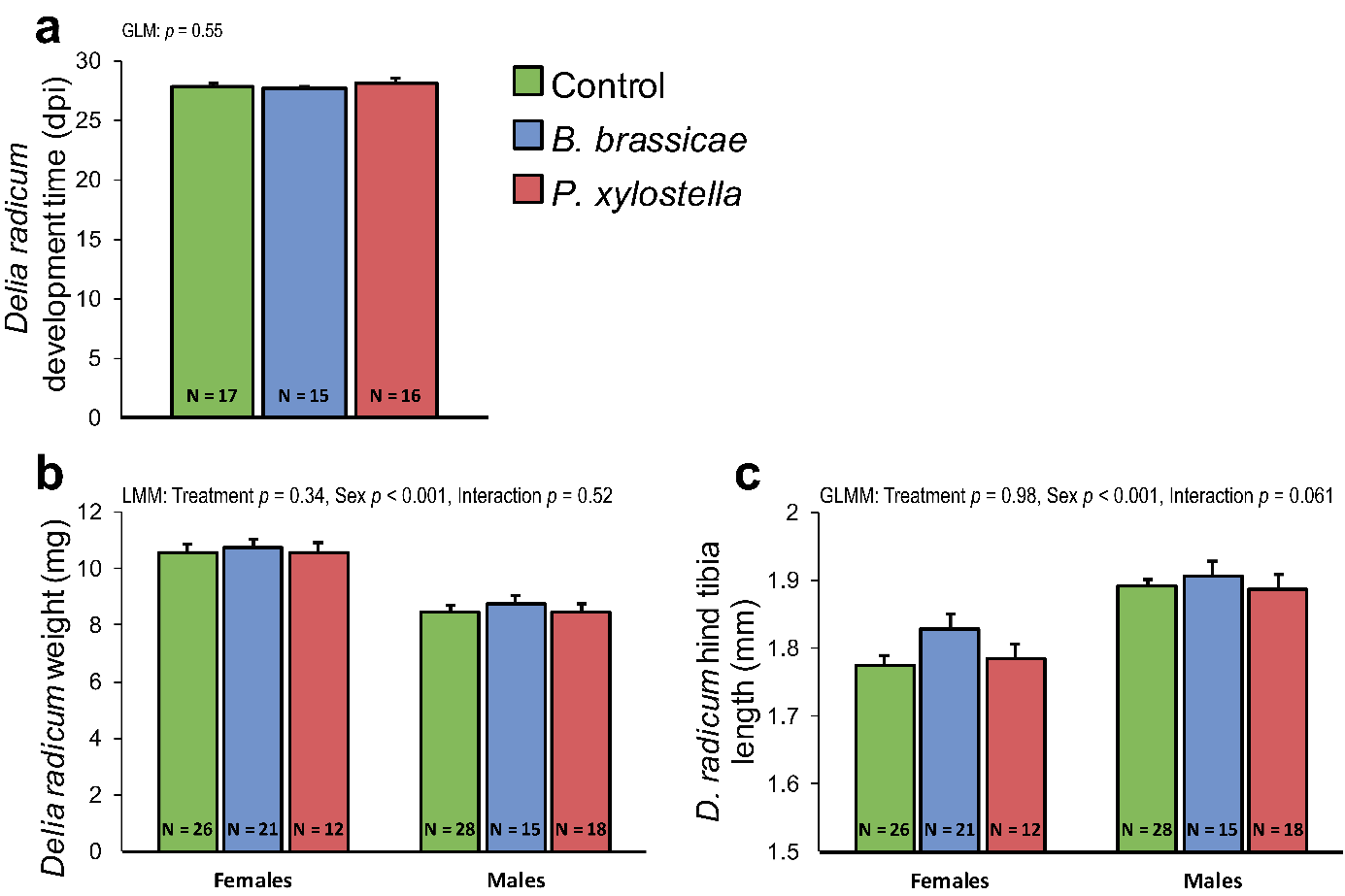


**Fig. S1**

Development time from neonate to adult (a), adult weight (b) and adult hind tibia length (c) of *Delia radicum* on *Brassica* *oleracea* var. *gemmifera* plants. Two days prior to *D. radicum* infestation, plants were induced by either *Plutella* *xylostella* or *Brevicoryne* *brassicae* on the leaves. Error bars indicate standard errors of the mean.


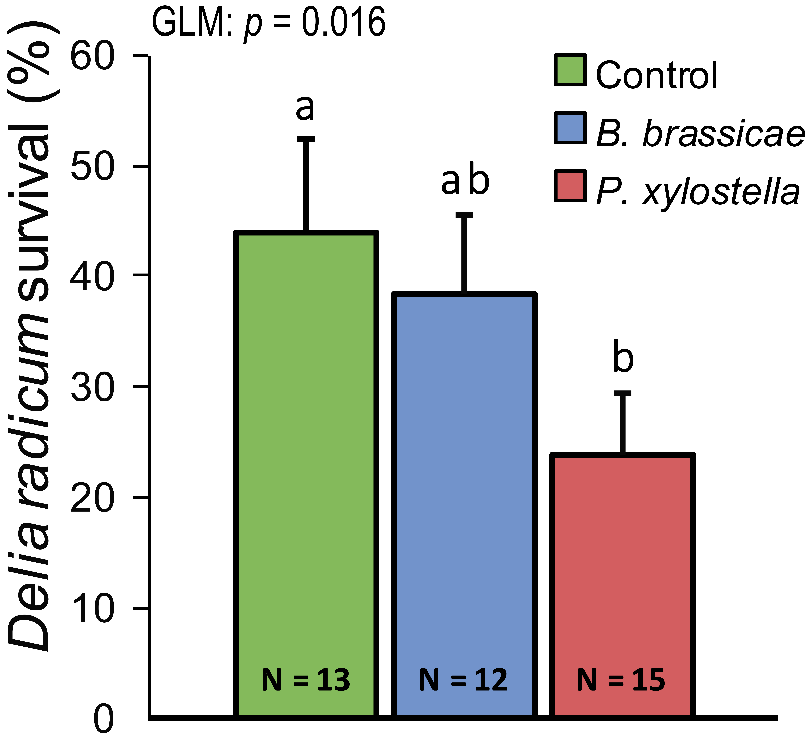


**Fig. S2**

Survival of *Delia radicum* flies to adulthood on *Brassica oleracea* var. *gemmifera* plants. Prior to D. radicum infestation, plants were induced by either *Plutella* *xylostella* or *Brevicoryne* *brassicae*. Methods similar as described in material and methods, with two exceptions: aboveground inducers were placed on the plant 7 days before *D. radicum* and left for 6 days, and 10 *D. radicum* larvae were used. Error bars indicate standard errors of the mean. Different letters indicate statistically significant differences (Tukey’s LSD, p < 0.05).


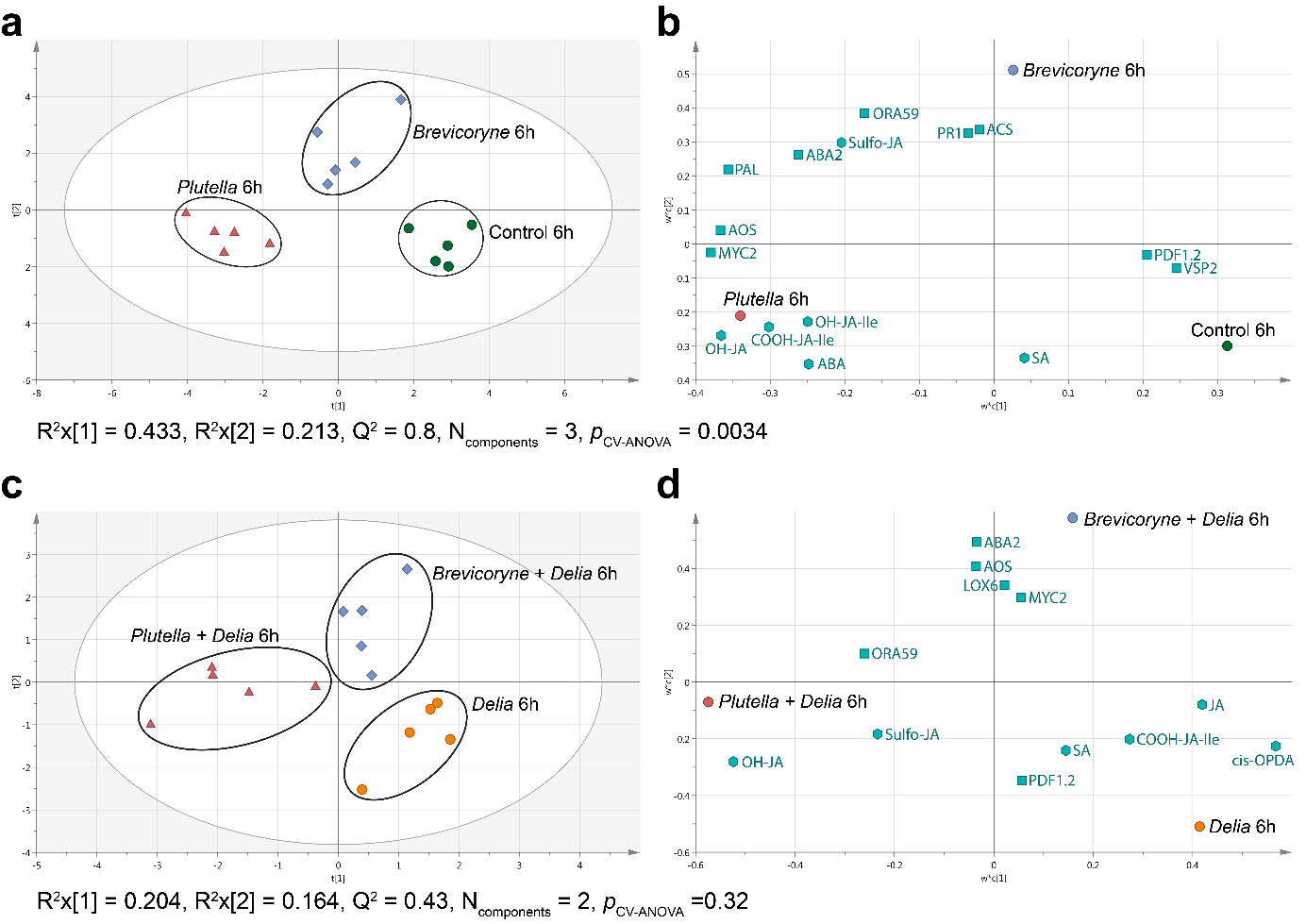


**Fig. S3**

PLS-DA analyses illustrating the defence response of *Brassica* *oleracea* primary roots to *Delia* *radicum* and two aboveground herbivores in terms of defence related genes and metabolites. Score plots (a,c,e) show separation of samples based on the PLS-DA model, loading plots (b,d,f) show the contribution of each gene/metabolite included in the model. The first model (a,b) shows differences between the response of primary roots to different aboveground herbivores in the absence of root herbivory. The second model (c,d) shows how primary roots respond to *D. radicum* in the presence of aboveground herbivores. Final models were generated by discarding the least important genes/metabolites from full models (VIP < 0.75). Both models were made using only one time point, 6 h after *D. radicum* infestation. Aboveground treatments are indicated by shapes, circles: no aboveground herbivores, triangles: *Plutella xylostella*, diamonds: *Brevicoryne* *brassicae*. Grey ellipses in score plots indicate Hotelling’s T2 (95%). Black circles delineate treatment groups, they have no statistical value. In loading plots, squares show genes and hexagons show metabolites.

#
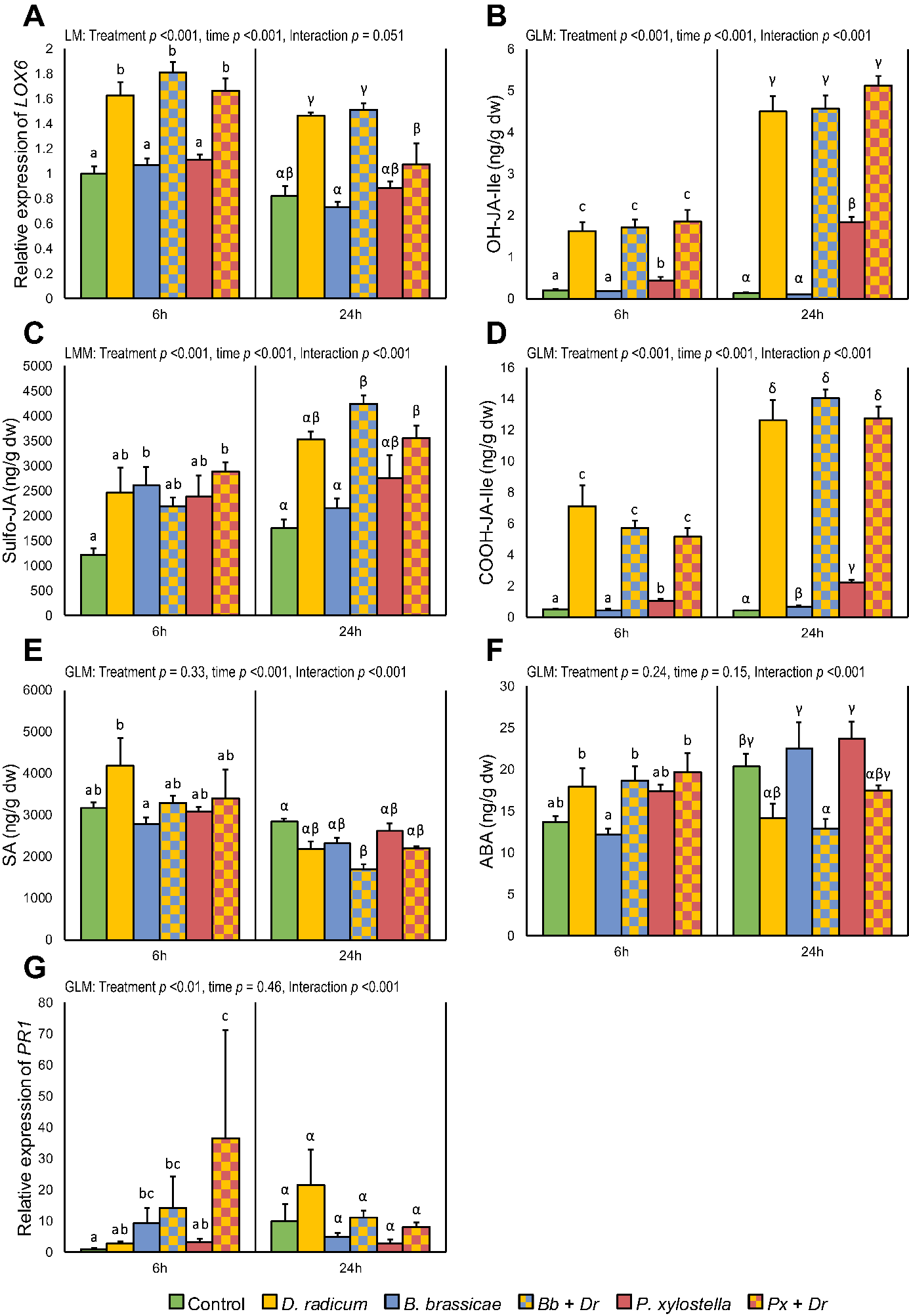


**Fig. S4**

Expression of genes and concentrations of metabolites related to defence signalling in primary roots of *Brassica* *oleracea* var. *gemmifera* plants induced by aboveground (*Brevicoryne* *brassicae* or *Plutella* *xylostella*) and belowground (*Delia* *radicum*) insect herbivores. Time points indicate time since *D. radicum* induction, plants were infested with aboveground herbivores 48 h prior to this. Error bars indicate standard errors of the mean, N = 5, each sample represents 3 pooled plants. Different letters indicate statistically significant differences between treatments within a time point (Tukey’s LSD, p < 0.05).


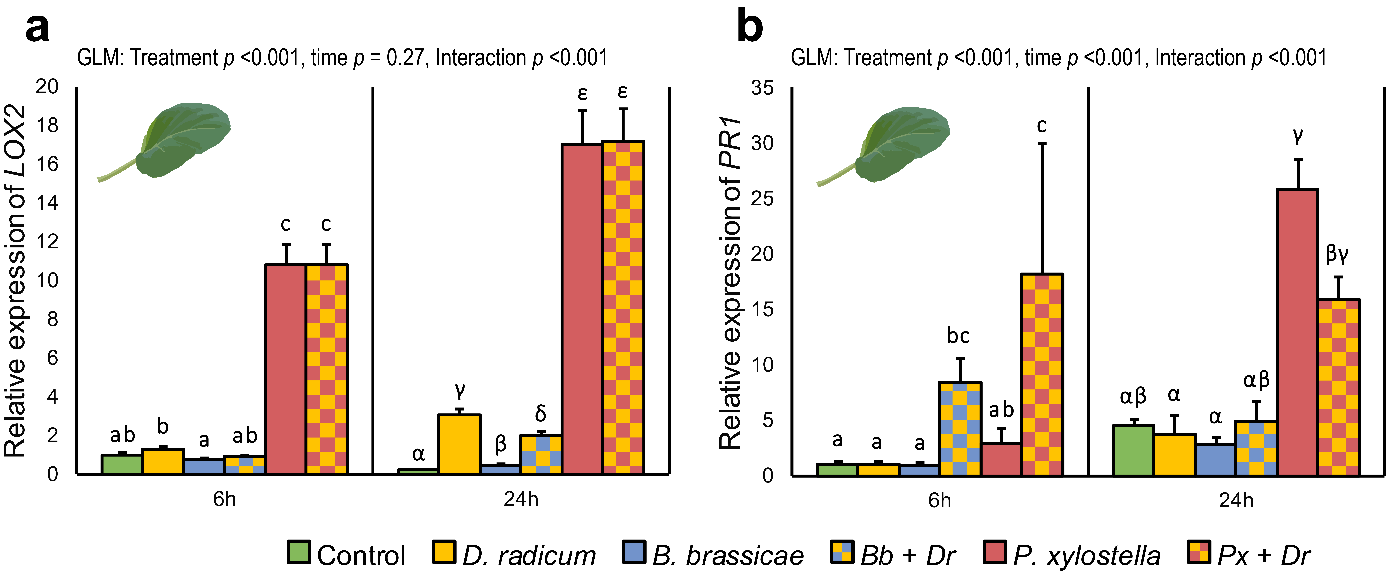


**Fig. S5**

Expression of *LOX2* (a) and *PR1* (b) in leaves of *Brassica* *oleracea* var. *gemmifera* plants induced by aboveground (*Brevicoryne* *brassicae* and *Plutella* *xylostella*) and belowground (*Delia* *radicum*) insect herbivores. Samples were taken at the site of leaf damage. Time points indicate time since *D. radicum* induction, plants were infested with aboveground herbivores 48 h prior to this. Error bars indicate standard errors of the mean, N = 5, each sample represents 3 pooled plants. Different letters indicate statistically significant differences between treatments within a time point (Tukey’s LSD, p < 0.05).

**Table S1**

Primers used for qPCR analyses of *Brassica oleracea* roots and leaves.

| **qPCR primers for *B. oleracea* roots** | |  |
| --- | --- | --- |
| Gene name | Forward primer ('5 to '3) | Reverse primer ('5 to '3) |
| PR1 | GTCAACGAGAAGGCTAACTATAACTACG | TTACACCTTGCTTTGCCACATCC |
| MYC2 | GGCTGGACCTACGCTATATTCTGG | AGAAAAACCACTCCGTATCCGT |
| ACS | ACTACGGTTGGCTGAAAGAC | GAGAAACGTTCAGCTTCACC |
| PAL | TCGCTATGGCTTCTTACTGCTCTG | GAGGTCTTACGAGATGAGATGAGTCC |
| VSP2 | GACTATCTCACTTCCCCACAG | CGGGTCTAT CTTCTCTGTCC |
| LOX6 | AGGAGCTGCCAATTCGAAGC | CGCCTGTTCCAAAGTCATTCCA |
| AOS | ACCGCTTGCGACTAGGGATC | CAAAGTCCTTACCGGCGCAC |
| ABA2 | GCATCGCTCGTCTGTTCCAC | CGGCGAAGTCAACAGCGTTA |
| ORA59 | AGGAAAGGGATAAGAGTGTGGCT | TCAAAGCTATCACCGGAGACTC |
| PDF1.2 | CTCTCGAAGCACCAACAATG | CCATGTCGTGCTTTCTCAAGG |
| **qPCR primers for *B. oleracea* leaves** | |  |
| Gene name | Forward primer ('5 to '3) | Reverse primer ('5 to '3) |
| LOX2 | GCCATTGAGTTGACTCGTCC | GGATGCATGGCACTTAGTTGT |
| PR1 | GTCAACGAGAAGGCTAACTATAACTACG | TTACACCTTGCTTTGCCACATCC |
| **qPCR primers for *B. oleracea* reference genes** | | |
| Gene name | Forward primer ('5 to '3) | Reverse primer ('5 to '3) |
| GADPH | GCTACGCAGAAGACAGTTGATGG | TGGGCACACGGAAGGACATAC |
| Act-2 | ACATTGTGCTCAGTGGTGGA | TCTGCTGGAATGTGCTGAGG |
| Btub | GTCAAGTCCAGCGTCTGTGA | TCACACGCCTGAACATCTCC |
| EF1a | GGTACCTCCCAGGCTGATTG | TCAGGTAKGAAGACACCTCCTTG |
| PER4 | TATCCTCTGCAGCCTCCTCA | ACACACAGACTGAAGCGTCC |
| SAR1a | ATCTCTAGCCACCGTTCCCT | TTCCTGACGATGCTGCACAT |
